# Promoting the Integration of Elderly Healthcare and Elderly Nursing: Evidence from the Chinese Government

**DOI:** 10.3390/ijerph192416379

**Published:** 2022-12-07

**Authors:** Mo Hu, Zhiyuan Hao, Yinrui Yin

**Affiliations:** 1 School of Journalism and Communication, Nanjing Normal University, Nanjing 210024, China; 2 School of Business and Management, Jilin University, Changchun 130012, China; 3 School of Mathematics and Sciences, Nanjing Normal University, Nanjing 210046, China

**Keywords:** healthy aging, elderly healthcare, elderly nursing, health promotion, government policy, information collaboration network

## Abstract

The increase of the aging population in China and the rise of the concept of healthy aging have accelerated the transformation and upgrading of the traditional elderly nursing pattern. Nevertheless, there is a critical limitation existing in the current situation of China’s elderly care, i.e., the medical institutions do not support elderly nursing and the elderly nursing institutions do not facilitate access to medical care. To eliminate the adverse impact of this issue, twelve ministries and commissions of the Chinese government have jointly issued a document, i.e., the Several Opinions on Further Promoting the Development of Combining the Healthcare with the Elderly care (SOFPDCHE), to provide guidance from the government level for further promoting the integration of elderly healthcare and elderly nursing. Under this background, this paper constructs a healthcare–nursing information collaboration network (HnICN) based on the SOFPDCHE, proposing three novel strategies to explore the different roles and collaboration relationships of relevant government departments and public organizations in this integration process, i.e., the node identification strategy (NIS), the local adjacency subgroup strategy (LASS), and the information collaboration effect measurement strategy (ICEMS). Furthermore, this paper retrieves 484 valid policy documents related to “the integration of elderly healthcare and elderly nursing” as data samples on the official websites of 12 sponsored ministries and commissions, and finally confirms 22 government departments and public organizations as the network nodes based on these obtained documents, such as the National Health Commission of the People’s Republic of China (NHC), the Ministry of Industry and Information Technology of the People’s Republic of China (MIIT), and the National Working Commission on Aging (NWCA). In terms of the collaboration effect, the results of all node-pairs in the HnICN are significantly different, where the collaboration effect between the NHC and MIIT is best and that between the NATCM and MIIT is second best, which are 84.572% and 20.275%, respectively. This study provides the quantifiable results of the information collaboration degree between different government agencies and forms the optimization scheme for the current collaboration status based on these results, which play a positive role in integrating elderly healthcare and elderly nursing and eventually achieving healthy aging.

## 1. Introduction

The aging of the population has become one of the serious social issues in the world [1,2,3,4], and in addition, elderly nursing and elderly healthcare cannot be ignored in the stable and harmonious development process of a society [5,6,7,8,9]. With the degree of China’s population aging exacerbating and the elderly population base increasing, the issues of elderly nursing and elderly healthcare in China have also received increasing research attention by the worldwide investigators. To date, there are various studies focusing on the aforementioned issues, such as constructing a physical and medical care integrated model for the elderly in the community [10], analyzing the health transition of the elderly [11], researching the supply and demand of elderly care service resources [12], exploring the elderly care social enterprises [13], and identifying the status of successful aging and the factors influencing empty-nest elderly in China [14].

For a long time, elderly nursing and healthcare have been two relatively independent areas in China, which could inevitably cause a situation of information disparity, i.e., the elderly nursing information obtained by healthcare institutions and the elderly healthcare information obtained by elderly nursing institutions are inconsistent. How to coordinate the relationship between elderly nursing and healthcare has become a crucial segment for promoting the realization of China’s healthy aging. To this end, numerous researchers have conducted relevant studies from different perspectives. For example, Zhou et al. reviewed China’s medical and old-age care integration model to promote the development of medical care-integrated old-age care [15], while Wei et al. conducted an investigation on elderly people’s willingness to combine medical care and healthcare and identify the related factors [16]. Similarly, Yuan et al. conducted a qualitative study in Anhui (in the middle area) and Fujian Province (in the eastern area) to explore the integrated care for the aged population [17]. Nevertheless, there is still a limitation existing in the aforementioned studies, i.e., these findings cannot effectively and accurately measure the role of different government departments and public organizations in promoting the integration of elderly healthcare and elderly nursing, which means they cannot provide explicit data references for these departments and organizations to optimize the current elderly nursing pattern. Therefore, exploring the different roles of relevant departments and organizations in this integration process under the quantitative perspective is a feasible motivation for auxiliary healthy aging.

In fact, promoting the healthcare–nursing integration not only requires the full cooperation between medical institutions and elderly nursing institutions, but also needs strong support from multiple industries and fields, where the government plays the role of top-level design in this process. To further promote the integration of elderly healthcare and elderly nursing from the government level, 12 ministries and commissions of the Chinese government jointly issued a document in October 2019, i.e., the Several Opinions on Further Promoting the Development of Combining the Healthcare with the Elderly care (SOFPDCHE). Notably, different government agencies have different administrative functions, and in that case, the integration process will inevitably involve multiple functional departments, and the collaboration between the different departments and organizations definitely plays an important role in promoting the integration process. Furthermore, the collaboration content between different functional departments is mainly reflected in the relevant information, i.e., the information about the integration of elderly healthcare and elderly nursing. Therefore, in this paper, we conduct the whole study under the perspective of information collaboration between different government agencies, constructs a healthcare–nursing information collaboration network (HnICN) [18,19] based on the SOFPDCHE, and comprehensively explores the inner features of the HnICN by analyzing the global structure, calculating the attribute variables of the adjacency subgroups, and measuring the collaboration effects of the node-pairs. The main contributions and innovations of this study are highlighted as follows:

(1) To promote the integration of elderly nursing and elderly healthcare in China, we propose three novel strategies in the HnICN, i.e., the node identification strategy (NIS), the local adjacency subgroup strategy (LASS), and the information collaboration effect measurement strategy (ICEMS).

(2) To explore the different roles of network nodes in the HnICN, we retrieve 484 valid policy documents related to “the integration of elderly healthcare and elderly nursing” as data samples on the official websites of 12 sponsored ministries and commissions, and finally confirm 22 government departments and public organizations as the network nodes based on these obtained documents.

(3) To provide the explicit data references for optimizing the current elderly nursing pattern, we conduct an exploration analysis of the HnICN under a quantitative perspective.

## 2. Research Framework and Data Description

### 2.1. Research Framework

In this paper, the experimental analysis was conducted via the following steps:

Step 1: Collecting the relevant government policy documents as the data samples based on the SOFPDCHE and determining the basic node composition of the HnICN.

Step 2: Introducing the named entity recognition method, the optimized dictionary, and the social network analysis theory to design the NIS for analyzing the specific network nodes and the corresponding relationships between different nodes.

Step 3: Constructing the LASS to clarify the subgroup features of the HnICN, including calculating the index weight, the node weight, and the collaboration coverage of each node.

Step 4: Designing the ICEMS based on the collaboration coverage result of each node to calculate the collaboration structure entropy and eventually calculate the collaboration effect values of the node-pairs.

**Figure 1:**
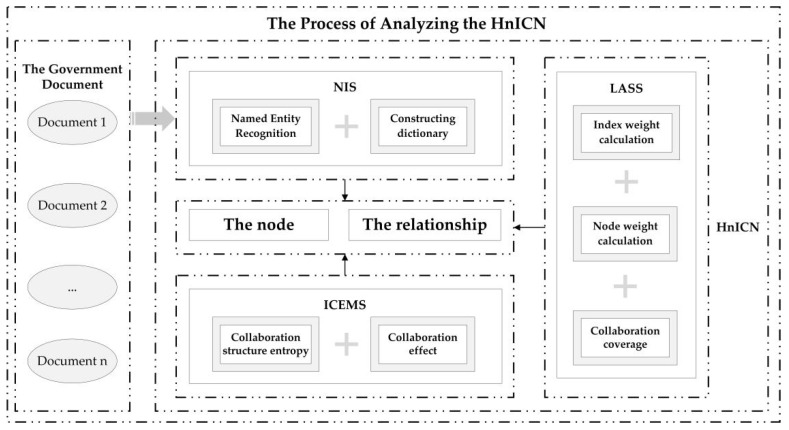
The process of analyzing the HnICN.

The research process of this study is shown in Figure 1.

### 2.2. Data Description

As mentioned above, promoting the integration of elderly healthcare and elderly nursing is a significant policy to realize healthy aging in China, where the governments play the role of top-level design in this process. The top-level design role of governments is mainly reflected in the relevant policy documents and measures which are issued by different government agencies; in other words, these relevant policy documents and measures are the target sources of data samples in this study.

In addition, the HnICN constructed in this paper is an information collaboration network, which is a social network in essence, where the network nodes and node relationships are both significant attributes. Therefore, we took the government agencies which are mentioned in the collected documents as the network nodes and considered that a relationship of any two network nodes is the connection between these two government agencies. Notably, only when two government agencies are mentioned together in the same document do we consider a connection has arisen between these two agencies.

To ensure the credibility and authority of the obtained data, we confirmed the data samples based on the following three significant principles, i.e., the relevance of policy documents, the reasonable time span, and the target government departments. The specific descriptions are shown as follows:

- In terms of the policy documents, we took the SOFPDCHE as the base guidance document and retrieved the policy documents related to “the integration of elderly healthcare and elderly nursing” on the official websites of the 12 sponsored ministries and commissions as the data samples, including the ministerial (committee) orders, the public announcements, and the notices.
- In terms of the time span, the starting time for document retrieval was 23 October 2019, which is the issued time of SOFPDCHE, and the deadline for document retrieval was 1 February 2022.
- In terms of the target government departments, because each policy document involves a number of different government departments and public organizations, we obtained the target departments and organizations after conducting the statistics and duplicates’ elimination for the institution names which are mentioned in the obtained documents.

Finally, we collected 484 valid policy documents to conduct the subsequent experiments and confirmed 22 relevant government departments and public organizations as the network nodes of the HnICN. The characteristics of these departments and organizations are shown in Table 1.

## 3. The Healthcare–Nursing Information Collaboration Network (HnICN)

In this paper, we mainly focus on exploring the roles of different government departments and public organizations in promoting the integration of elderly nursing and elderly healthcare. To this end, we constructed the HnICN and explored the connections between different network nodes under a quantitative analysis perspective. In particular, there are three significant strategies in analyzing the HnICN, i.e., the node identification strategy (NIS), the local adjacency subgroup strategy (LASS), and the information collaboration effect measurement strategy (ICEMS), where adopting the NIS can identify the important department nodes and the connections between different nodes, while adopting the LASS can obtain the attribute characteristics of the subgroups. In addition, the ICEMS aims to measure the collaboration effects of different node-pairs, which is the most significant part in the HnICN. The specific details of these three strategies are described as follows.

### 3.1. The Node Identification Strategy (NIS)

As mentioned above, this paper mainly focuses on optimizing the information collaboration status between different government departments and public organizations for promoting the integration of elderly healthcare and elderly nursing by exploring the HnICN, which means that if we can analyze the information connections existing in the different nodes, identify the important node in the current network, and eventually conclude the corresponding regularity, we will provide valid decision supports to promote healthy aging. To achieve this research purpose, we searched for the powerful evidence in the relevant policy documents issued by China’s government. Therefore, we adopted the named entity recognition method [20,21] to identify the network nodes of the HnICN, i.e., the government departments and public organizations mentioned in the relevant documents, and utilized the social network analysis [22,23] to explore the relationships between different nodes.

**Figure 2:**
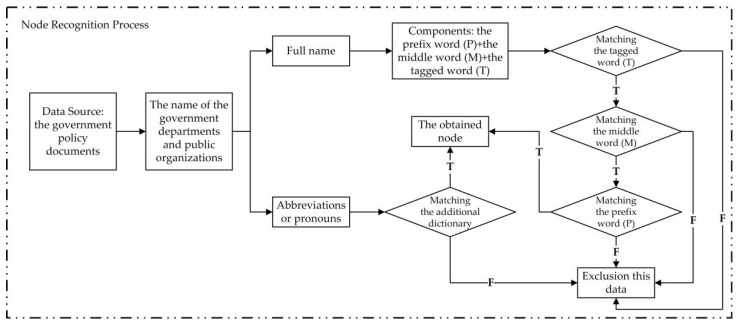
The node recognition process.

In terms of the node recognition, the method utilized in this section comprehensively integrates multiple recognition dimensions, such as the word frequency, the part of speech, and the length of the word, which divides the names of China’s government departments and public organizations into three parts, i.e., the prefix word (P), the middle word (M), and the tagged word (T), where the tagged word (T) is the core part in the recognition process. In addition, because many pronouns and abbreviations can be used to denote these government departments and public organizations in the common expressions, this paper further optimizes the original dictionary used in the named entity recognition process by collecting and adding theses corresponding pronouns and abbreviations [24,25,26]. The node recognition processes of the NIS are shown in Figure 2.

In terms of relationship recognition, the existence of information connections between any two identified nodes means that there are some relationships between these two nodes. For example, if two different departments or organizations are mentioned in the same document, it indicates that the promotion of this matter requires the joint participation of these two institutions; in other words, an information collaboration relationship has been generated between these two institutions. Notably, the node importance is a significant attribute, which has a great impact on the node relationships in the HnICN. Therefore, we adopted the degree centrality of the social network analysis to elaborate the importance of different nodes, where the calculation process of degree centrality is as follows:

(1) $${degree\_cen}_{i}=\sum_{j=1,j\neq i}^{n}connection\left(i,\;j\right)$$

In Equation (1), the *degree_cen_i_* indicates the degree centrality of the nodes, the *i* and the *j* are two different nodes, the *n* is the number of nodes, and the value of *connection(i, j)* could be taken as 0 or 1, where if there is a connection between the node *i* and node *j*, the *connection(i, j)* is taken as 1, otherwise, the *connection(i, j)* is taken as 0.

### 3.2. The Local Adjacency Subgroup Strategy (LASS)

Each node involves a different number of connections in the HnICN, so the network structure of HnICN is a topology with different densities for each node [27,28], where the density indicates the potential collaboration range of each node. In fact, the information collaboration process related to a certain matter may require multiple departments’ participation. For example, we assume that the completion of a certain matter needs the participation of department 1, department 2, department 3, and department 4, containing two information transmission processes, i.e., the “department 1→department 2→department 3” and the “department 4→department 3”, which shows that these 4 departments have different collaboration ranges and play different roles in the collaboration processes. In that case, if we can obtain the collaboration range of different nodes, we will discover the pathway for optimizing the structure of the HnICN. Therefore, we designed the LASS to construct subgroups and proposed the concept of collaboration coverage. Notably, the LASS adopts a clustering idea [29,30] to transform the relationship between the nodes into that between the subgroups [29], where each node could be selected as a center of a subgroup and each subgroup can be formed by other non-central nodes which all have connections with the selected center node.

As mentioned above, the subgroup is a significant concept to deeply describe the role that a node plays in the HnICN; moreover, the collaboration coverage results could be obtained based on the subgroups. Specifically, there are two important attribute variables in the subgroup, i.e., the index weight and the node weight. In terms of the index weight, because the analyzed data samples are the government documents, where the target information about the integration of elderly healthcare and elderly nursing is mainly focused on a few fixed aspects, we should transform these aspects into a few analyzable indexes. In fact, the number of departments or organizations involved in each aspect is different, which means the analyzable indexes could have some impacts on the role of different nodes played in the HnICN. Therefore, we need to calculate the index weight in the subgroups. According to the guiding ideology of the SOFPDCHE and the related literature [31,32,33,34], we eventually concluded five important indexes, i.e., the main policies, the medical resources, the natural resources, the economic support, and the personnel training and employment encouragement. Meanwhile, we adopted the entropy method [35,36,37] to measure the weight values of these five indexes. The specific calculation processes of the index weight are shown as follows:

(2) $$p\left(i,\;j\right)=\frac{data\left(i,\;j\right)}{\sum_{i=1}^{n}data\left(i,\;j\right)}$$

(3) $${inf\_entropy}_{j}=-\frac{1}{ln\left(n\right)}\times \sum_{i=1}^{n}p\left(i,\;j\right)\times ln\left(p\left(i,\;j\right)\right)$$

(4) $${inf\_utility}_{j}=1-{inf\_entropy}_{j}$$

(5) $${weight\_index}_{j}=\frac{{inf\_utility}_{j}}{\sum_{j=1}^{k}\left({inf\_utility}_{j}\right)}$$

Equations (2)–(5) mainly focus on the weight computing of the five aforementioned indexes, where the *data(i, j)* indicates the number of mentions of the *i*th department in the *j*th index aspect (in this paper, the *data(i, j)* is the results after normalization, and the value range is from 1.0 to 2.0), the *p(i, j)* indicates the proportion of the *j*th index among all indexes which are related to the *i*th department, the *n* is the total number of department nodes, the *inf_entropy_j_* and the *inf_utility_j_* are the information entropy of the *j*th index and the information utility of the *j*th index, respectively, and the *weight_index_j_* is the final weight result of the *j*th index.

In terms of the node weight, we introduced the TOPSIS method [38,39,40] and measured the node weight of different nodes based on the index weight calculation results, which are shown as follows:

(6) $$Z\left(i,\;j\right)=\frac{data\left(i,\;j\right)-min\left(data\left(j\right)\right)}{max\left(data\left(j\right)\right)-min\left(data\left(j\right)\right)}\times weight\_index_{j}$$

(7) $$Z_{j}^{+}=\left\{{max}_{i=1}^{n}\left(Z\left(i,\;1\right)\right),\;\ldots ,\;{max}_{i=1}^{n}\left(Z\left(i,\;j\right)\right)\right\}$$

(8) $$Z_{j}^{-}=\left\{{min}_{i=1}^{n}\left(Z\left(i,\;1\right)\right),\;\ldots ,\;{min}_{i=1}^{n}\left(Z\left(i,\;j\right)\right)\right\}$$

(9) $${dis}_{i}^{+}=\sqrt{\sum_{j=1}^{m}{\left(Z\left(i,\;j\right)-Z_{j}^{+}\right)}^{2}}$$

(10) $${dis}_{i}^{-}=\sqrt{\sum_{j=1}^{m}{\left(Z\left(i,\;j\right)-Z{\left(i,\;j\right)}_{j}^{-}\right)}^{2}}$$

(11) $${close}_{i}=\frac{dis_{i}^{-}}{dis_{i}^{-}+dis_{i}^{+}}$$

(12) $${weight\_node}_{i}=\frac{close_{i}}{\sum_{o=1}^{n}close_{o}}$$

Equations (6)–(12) aim to describe the computing process of the node weight, where the *Z(i, j)* is a normalization matrix, and the *min(data(j))*, the *max(data(j))*, the ${max}_{i=1}^{n}$*(Z(i, j))*, and the ${min}_{i=1}^{n}$*(Z(i, j))* indicate the minimum value of the *j*th index, the maximum value of the *j*th index, the maximum value of each column in the matrix *Z(i, j),* and the minimum value of each column in the matrix *Z(i, j)*, respectively. In addition, the ${dis}_{i}^{+}$, the ${dis}_{i}^{-}$, and the *close_i_* represent the distance from the best solution, the distance from the worst solution, and the relative closeness results, where the *m* indicates the number of indexes.

In addition, inspired by the literature [41,42], we adopted an average connection distribution of a certain subgroup to calculate the collaboration coverage of this node, where the collaboration coverage denotes the potential collaboration range of a certain node, which emphasizes the node’s collaboration ability. Therefore, the collaboration coverage can be calculated as in Equation (13):

(13) $${\mathrm{collaboration}\_\mathrm{coverage}}_{i}=adj\_par\times \frac{\sum_{i=1}^{n}weight\_node_{i}\times count\left(subgroup{\left(i\right)}_{j},\;center_{i}\right)}{amount\left(subgroup\left(i\right)\right)-1}$$

where the meaning of the *adj_par* is an adjust parameter which can be set manually (we set the value of the *adj_par* to 10), the *count(subgroup(i)_j_, center_i_)* indicates the number of connections between the non-center nodes and center node *i*, the *subgroup(i)* indicates a subgroup which selects the node *i* as the center node, and the *amount(ψ)* is a counting function to calculate the number of all nodes in a subgroup.

### 3.3. The Information Collaboration Effect Measurement Strategy (ICEMS)

The NIS and the LASS can be utilized to analyze the HnICN under a global perspective and a local perspective, respectively. However, we still need to explore the inner relationships of a certain node-pair by analyzing the collaboration degree between these two nodes. Therefore, we proposed the ICEMS to measure the information collaboration effect between different nodes.

Generally, the information collaboration process requires at least two nodes to participate, which means the participation tendency or possibility of each node in the collaboration process is also an important factor to influence the collaboration results. In other words, if a node has a superior potential collaboration range but a low participation tendency, the corresponding collaboration processes which contain this node have difficulty achieving the desired results. In addition, the transmission object in the collaboration process is the information about the integration of elderly healthcare and elderly nursing; therefore, we introduced the information entropy theory [43,44] to design the collaboration structure entropy, which is used to describe the collaboration tendency of a node. The calculation process of the collaboration structure entropy is shown as Equation (14):

(14) $$\mathrm{cse}\left(i\right)={\mathrm{collaboration}\_\mathrm{coverage}}_{i}\times \log_{10}{\mathrm{collaboration}\_\mathrm{coverage}}_{i}$$

where the *cse(i)* is the collaboration structure entropy of node *i*. Based on the collaboration structure entropy, we could obtain the collaboration effect measurement process as follows:

(15) $$collaboration\_effect{\left(i,\;j\right)}_{i\neq j}=\left\{\begin{array}{l} \frac{cse\left(i\right)\times cse\left(j\right)}{\max\left(cse\left(i\right)\times cse\left(j\right)\right)\left(1+\psi \right)}\;\;\;\;cse\left(i\right)\times cse\left(j\right)=\max\left(cse\left(i\right)\times cse\left(j\right)\right)\\ \frac{cse\left(i\right)\times cse\left(j\right)}{\max\left(cse\left(i\right)\times cse\left(j\right)\right)}\;\;\;\;\;\;\;\;\;\;\;cse\left(i\right)\times cse\left(j\right)\neq \max\left(cse\left(i\right)\times cse\left(j\right)\right) \end{array}\right.\;$$

where the calculation process contains two situations for avoiding the maximum value reaching 100% and making the calculation results within the percentage range of 0–100% (in this paper, the parameter *ψ* was set to 10,000).

## 4. Results

This section mainly presents the result analyses of the NIS, the LASS, and the ICEMS, where the NIS aims to elaborate the global structure of the HnICN by analyzing the relationships of different node-pairs and the importance of different nodes, the LASS was used to describe the local structure of the HnICN by constructing the adjacent subgroups and calculating the collaboration coverage, and the ICEMS mainly focuses on the quantification calculation of the information collaboration effect.

### 4.1. The Nodes and Relationships

**Figure 3:**
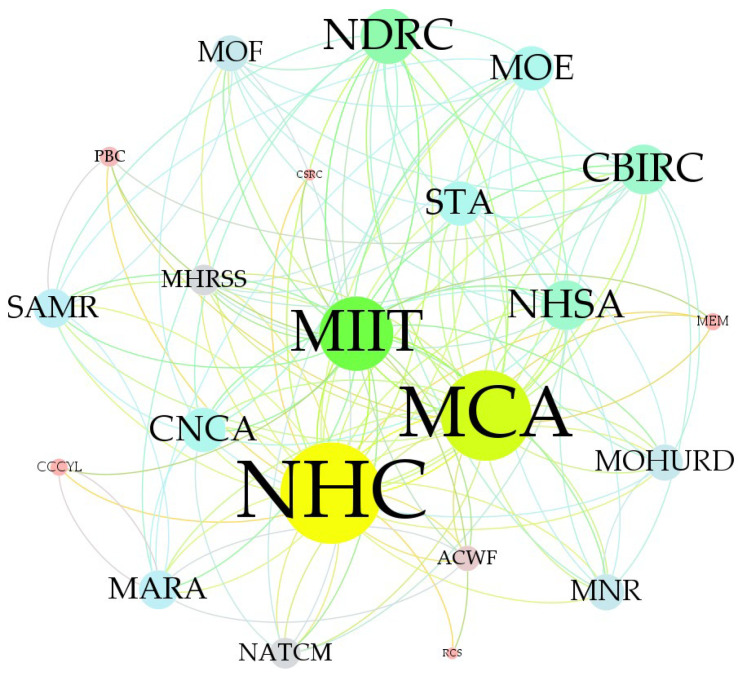
The global visualization result of the HnICN.

**Figure 4:**
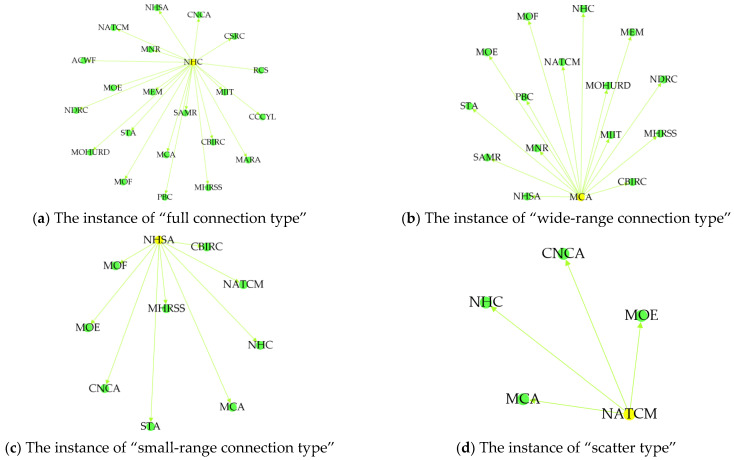
The node relationship figure of the out-degree. (**a**) The representative result of “full connection type” (taking the NHC node as an example), (**b**) the representative result of “wide-range connection type” (taking the MCA node as an example), (**c**) the representative result of “small-range connection type” (taking the NHSA node as an example), and (**d**) the representative result of “scatter type” (taking the NATCM node as an example).

**Figure 5:**
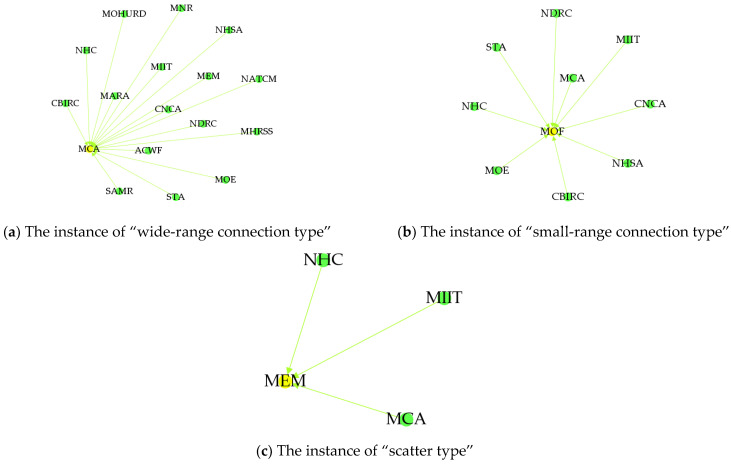
The node relationship figure of the in-degree. (**a**) The representative result of “wide-range connection type” (taking the MCA node as an example), (**b**) the representative result of “small-range connection type” (taking the MOF node as an example), and (**c**) the representative result of “scatter type” (taking the MEM node as an example).

We adopted the Gephi software (the version is 0.9.7) to visualize and analyze the network nodes and the relationships of node-pairs, which are shown in Table 2 and Table 3 and Figure 3, Figure 4 and Figure 5.

In Figure 3, the circle indicates the government department node in the HnICN, and the line segment between any two circles denotes the connection of these two nodes. Notably, there are four different node relationship categories in the HnICN, i.e., the full connection type, the wide-range connection type, the small-range connection type, and the scatter type, where these categories are divided based on the results of degree centrality. Specifically, we defined that the degree centrality of the full connection type is greater than 20 or equal to 20, the degree centrality value range of the wide-range connection type is from 10 to 20, the degree centrality value range of the small-range connection type is from 5 to 10, while the degree centrality of the scatter type is less than 5. In fact, according to the Table 2 and Table 3 and the Figure 4 and Figure 5, we can find that the node importance results in this network are different and the collaboration distributions in the global structure are unbalanced. Particularly, for the out-degree, the number of nodes with the full connection type was only 2, the number of nodes with the wide-range connection type was only 3, while the number of nodes with scatter type can reach 10. Meanwhile, for the in-degree, the number of nodes with the full connection type was 0, the number of nodes with the wide-range connection type was only 4, while the number of nodes with the small-range connection type can reach 12. Furthermore, we can find that the PBC, the CSRC, and the RCS all have no out-degree, but these three nodes all have in-degree, which means that these three departments and organizations are not the initiators or sponsors of relevant matters about promoting the integration of elderly healthcare and elderly nursing, but rather act as the task receivers.

### 4.2. The Calculation Results

Here, we present the corresponding calculation results of the LASS and the ICEMS, including the index weight results and the node weight results in the subgroups, the collaboration coverage results based on the subgroup, the collaboration structure entropy results of the 22 network nodes, and the final information collaboration effect results of different node-pairs. The specific results are shown in Table 4, Table 5, Table 6 and Table 7.

According to the Table 4, we can find that “the personnel training and employment encouragement” obtained the best weight value at 0.2599 among all 5 indexes, while “the main policies” obtained the second-best weight value at 0.2430, which indicates that most government departments and public organizations focus on these two aspects in promoting the integration of elderly healthcare and elderly nursing. Moreover, according to the Table 5, we found that the NHC node obtained the best weight value at 0.188 among the 22 nodes, while the MCA obtained the second-best weight value at 0.153, which indicates that these two nodes are more important than others in the current HnICN. Based on the node weight results, we obtained the collaboration coverage values and the collaboration structure entropy values, which can be seen in the Table 6.

In the Table 6, it is clearly shown that there are large differences among the 22 collaboration coverage values. According to these collaboration coverage values, we found that the MIIT and the NHC had far better results than the others; meanwhile, there are 16 nodes whose collaboration coverage values were less than 5. In addition, there are also significant differences among the 22 collaboration structure entropy values, where the largest value reached 237.84 while the smallest value was 0. However, the collaboration structure entropy value of 0 does not mean that there is absolutely no collaboration between these nodes but means that these nodes have a low tendency to participate in the collaboration process.

**Figure 6:**
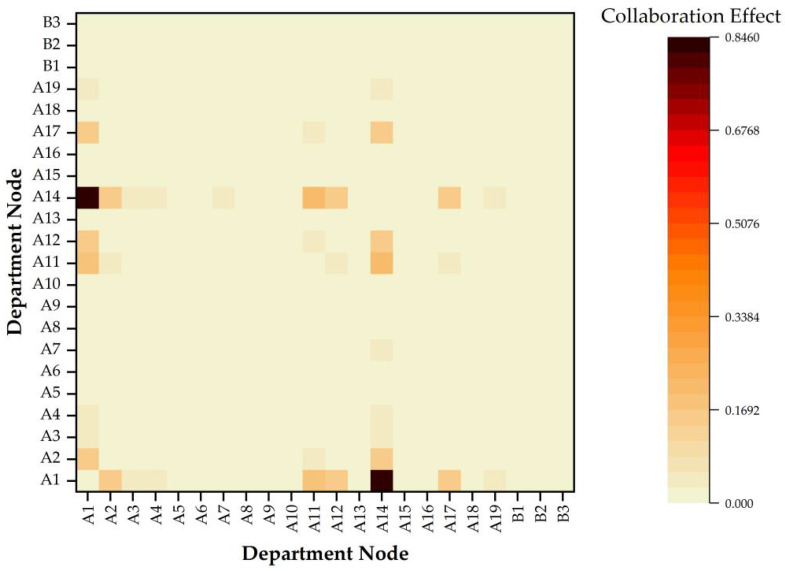
The information collaboration effect measurement results of different node-pairs.

Based on the collaboration structure entropy, the collaboration effect results can be calculated, which are shown in the Figure 6 and Table 7 (this table shows the top 10 information collaboration effect values and the corresponding node-pairs). In this paper, we utilized the collaboration effect to represent the collaboration degree between any two nodes. According to the Figure 6 and Table 7, it is clearly shown that the collaboration effects of different node-pairs in the current HnICN are particularly unbalanced, where the “A1–A14” obtained the best collaboration effect value at 84.572%, while the collaboration effect value of others were all less than 20.3%. Furthermore, the value of the “A3–A14” was only 4.964%, but it was ranked 10th among all values, which follows that there are slight collaboration effects between most government departments and public organizations in the current process of promoting the integration of elderly healthcare and elderly nursing.

### 4.3. Experimental Results’ Analysis

According to the obtained results of the NIS, we can conclude that the importance of different nodes in this information collaboration network decreased from the NHC (A1) to the RCS (B3). Notably, the node with the lowest importance was the RCS (B3), which is a public organization node, while the government department node with the lowest importance was the CSRC (A18). The possible reasons for this phenomenon are mainly reflected in the following two aspects: (i) The RCS mainly responds to the social aid and related services, but for the elderly groups in the current stage, the need of social aid is much smaller than the need of the integration of elderly healthcare and elderly nursing. (ii) Promoting the integration of elderly healthcare and elderly nursing requires government funding; however, the published policies and the proposed measures only belong to the top-level design in the whole process, which means there is not a specific collaboration measure between the CSRC and others in the current stage. Therefore, these two nodes had the lowest importance in the HnICN.

According to the obtained results of the LASS, we can conclude three significant findings about the role of the nodes. Firstly, in the current stage, the integration process of elderly healthcare and elderly nursing promoted by China’s government departments and public organizations revolves around the five aforementioned indexes, where “the personnel training and employment encouragement” and “the main policies” obtained the best weight value and the second-best weight value, respectively, which means the work focuses of the corresponding departments and organizations are on conducting the relevant policy-making and stimulating the development of relevant industries. In that case, the importance of some nodes related to these two aspects must be higher than others in the HnICN. Secondly, we obtained that the weight of different nodes in this information collaboration network also decreased from the NHC (A1) to the RCS (B3). However, because the collaboration process is affected by the aforementioned indexes, the node weight order is different from the degree centrality order. For example, the out-degree centrality of PBC was 0, which was ranked last, while the node weight value of PBC was 0.043, which was ranked 11th among all 22 nodes. The possible reason for this phenomenon is that the node weight indicates a collaboration potentiality of a certain node, while the degree centrality represents an importance in the current stage. Thirdly, it was clearly shown that the MIIT and the NHC had the highest collaboration coverage, which means the subgroups constructed by these two nodes had the largest collaboration range among all subgroups. In fact, the collaboration range is an important yardstick to determine the quality of the information collaboration process; in other words, if all nodes can obtain the larger collaboration range, more and more potential departments and organizations may participate in the collaboration process to promote the integration of elderly healthcare and elderly nursing, which can contribute to the realization of healthy aging.

According to the obtained results of the ICEMS, we can conclude that the collaboration effect between two nodes is positively correlated to the collaboration structure entropy value of these two nodes, and the collaboration effect values generated by the NHC node or the MIIT node collaborating with others could obtain a better rank relatively among all collaboration effect values. Specifically, the node-pair “A1–A14 (NHC–MIIT)” obtained the best collaboration effect value at 84.572%, while the second-best collaboration effect value obtained by the node-pair “A11–A14 (NATCM–MIIT)” was only 20.275%; furthermore, the values of the others were all less than 20%. Notably, the collaboration effect value of “A1–A14” was far higher than the others, and the possible reasons for this phenomenon are shown as follows: (i) China’s governments are currently promoting the achievement of the “digital China” policy and the “digital health” policy, where the MIIT is the most important government department in this process, and (ii) the medical information and health information of elderly groups is stored in the NHC. Therefore, the particularly strong collaboration effect of “NHC–MIIT” is destined to be generated based on these two situations.

## 5. Discussion

With the development of China’s elderly nursing industry, the government has been exploring the realization pathway of healthy aging. As a significant policy guideline, the integration of elderly healthcare and elderly nursing plays a vital role in achieving healthy aging in China. Nevertheless, there is still a critical limitation existing in the current stage, i.e., the collaboration status between different government departments and public organizations about promoting the integration of elderly healthcare and elderly nursing is unbalanced, which can retard the achievement of this goal. The reasons for the persistence of collaboration imbalance contain several aspects, such as the information collaboration relationships between different departments and organizations are indistinct, and the information collaboration effect cannot be effectively measured. In that case, if we can explore the specific information collaboration process and quantify the information collaboration effect, it will provide scientific and reliable decision support for promoting the healthcare–nursing integration process. Therefore, the HnICN constructed in this study has obvious theoretical and practical significance.

### 5.1. Theoretical Significance

In the context of the world’s population aging, the issues of elderly nursing and elderly healthcare have become a major research topic, where the existing studies have also discussed these issues from different perspectives, such as exploring the spatial accessibility of the elderly to healthcare services [45], establishing an integrated care link system in the healthcare industry for the elderly [46], investigating the demands for mobile internet-based home nursing services for the elderly [47], and analyzing the willingness of the elderly to choose nursing care [48]. Comparing with the existing studies, in this paper we proposed an exploratory approach to promote achieving healthy aging. On the one hand, under the information collaboration perspective, our investigation took the real policy documents of China’s government departments and public organizations as the data samples to explore the collaboration relationships and collaboration effects between different departments and organizations about promoting the integration of elderly healthcare and elderly nursing, which can provide a novel research perspective for other investigators to analyze the aforementioned issues, and can provide the reasonable theoretical guidance for relevant government officials to make the corresponding policies and measurements.

On the other hand, in the constructed HnICN, we introduced a quantification analysis idea to effectively calculate and analyze the node importance, the adjacent subgroups, the collaboration coverage values, the collaboration structure entropy, and the collaboration effect value, which are of great theoretical significance for finding the weaknesses in the current process of integrating the elderly healthcare and elderly nursing in China. Particularly, the information collaboration degree between any two nodes can be obtained by calculating the information collaboration effect, such as the collaboration degree between the MIIT and the NHC was 84.572%, and that between the MIIT and the NATCM was only 20.275%. These calculation results can effectively reflect the current information collaboration status between China’s government departments and public organizations, which are the important data references.

### 5.2. Practical Significance

Currently, the medical institution and elderly nursing institutions are independent of each other in China, which means the medical institutions do not support elderly nursing and the elderly nursing institutions do not facilitate access to medical care. This current situation not only increases the economic burden of families for supporting the elderly nursing, but also exacerbates the strain on medical resources. In that case, the practical significance of the HnICN can be manifested in the following two aspects:

In terms of the needs of elderly nursing, utilizing the HnICN can effectively improve the interactivity of the elderly healthcare information and the elderly nursing information, which can change the previous state of information dispersion for avoiding wasting information resources and can be helpful to better meet the needs of the elderly for healthy aging.

In terms of the macro-level role of the government, according to the calculation results of the information collaboration effect, the information collaboration process between different departments and organizations can be adjusted and optimized, and thus the role of different institutions in facilitating the integration of elderly healthcare and elderly nursing can be better carried out.

### 5.3. Limitations

In this study, the HnICN was constructed to further promote the realization of healthy aging. However, some limitations related to the HnICN should be noted:

(i) The volume of the data sample could be expanded. In this paper, although we collected 484 valid documents, these documents are limited to a certain time span and limited to be from the 12 sponsored ministries and commissions which have jointly issued the SOFPDCHE. Therefore, we will expand the source range of the documents and add more corresponding network nodes in the further research, which can lead to a more comprehensive analysis on the integration of elderly healthcare and elderly nursing.

(ii) The quantification method utilized in this paper could be improved. We conducted the quantification calculation mainly through the number of mentioned department names in a certain document; furthermore, the named entity recognition method adopted in this paper still has limitations in recognizing the pronouns or abbreviations of some government departments and public organizations. In the future, we will optimize the named entity recognition method to identify more valid department nodes and explore the other calculation indexes for measuring the information collaboration effect from the semantic perspective to enhance the rationality and reliability of the obtained results.

## 6. Conclusions

To promote the integration of elderly healthcare and elderly nursing, in this paper we utilized the HnICN to explore the different roles of the departments and organizations in this integration process. There were three significant strategies used in analyzing the HnICN, i.e., the NIS, the LASS, and the ICEMS, where the NIS aimed to elaborate the global structure of the HnICN, including analyzing the relationships of different node-pairs and the importance of different nodes, the LASS was used to describe the local structure of the HnICN by constructing the adjacent subgroups and calculating the collaboration coverage of the nodes, and the ICEMS mainly focused on the quantification calculation of the information collaboration effect. Based on this study, on the one hand, we will continue the in-depth research about the integration of elderly healthcare and elderly nursing for achieving a further breakthrough in promoting the healthy aging; on the other hand, we will specifically focus on analyzing the clinical resource redundancy or incompatibility between medical institutions and elderly nursing institutions.

## Figures and Tables

**Table 1:** The characteristics of the target government departments and public organizations.

Government Department	Coding	Official Websites (Accessed on 13 March 2022)
The National Health Commission of the People’s Republic of China (NHC)	A1	www.nhc.gov.cn
The Ministry of Civil Affairs of the People’s Republic of China (MCA)	A2	www.mca.gov.cn
The National Development and Reform Commission (NDRC)	A3	www.ndrc.gov.cn
The Ministry of Education of the People’s Republic of China (MOE)	A4	www.moe.gov.cn
The Ministry of Finance of the People’s Republic of China (MOF)	A5	www.mof.gov.cn
The Ministry of Human Resources and Social Security of the People’s Republic of China (MHRSS)	A6	www.mohrss.gov.cn
The Ministry of Natural Resources of the People’s Republic of China (MNR)	A7	www.mnr.gov.cn
The Ministry of Housing and Urban–Rural Development of the People’s Republic of China (MOHURD)	A8	www.mohurd.gov.cn
The State Administration for Market Regulation (SAMR)	A9	www.samr.gov.cn
The National Healthcare Security Administration (NHSA)	A10	www.nhsa.gov.cn
The National Administration of Traditional Chinese Medicine (NATCM)	A11	www.natcm.gov.cn
The China National Committee on Aging (CNCA)	A12	www.cncaprc.gov.cn
The Ministry of Agriculture and Rural Affairs of the People’s Republic of China (MARA)	A13	www.moa.gov.cn
The Ministry of Industry and Information Technology of the People’s Republic of China (MIIT)	A14	www.miit.gov.cn
The People’s Bank of China (PBC)	A15	www.pbc.gov.cn
The State Taxation Administration (STA)	A16	www.chinatax.gov.cn
The China Banking and Insurance Regulatory Commission (CBIRC)	A17	www.cbirc.gov.cn
The China Securities Regulatory Commission (CSRC)	A18	www.csrc.gov.cn
The Ministry of Emergency Management of the People’s Republic of China (MEM)	A19	www.mem.gov.cn
The Central Committee of the Communist Youth League of China (CCCYL)	B1	www.gqt.org.cn
The All-China Women’s Federation (ACWF)	B2	www.women.org.cn
The Red Cross Society of China (RCS)	B3	www.redcross.org.cn

**Table 2:** The out-degree centrality values of different nodes.

Category	Government Node	Coding	Out-Degree Centrality
Full Connection Type	NHC	A1	21
	MIIT	A14	20
Wide-range Connection Type	MCA	A2	15
	STA	A16	10
	CBIRC	A17	10
Small-range Connection Type	NHSA	A10	9
	NDRC	A3	7
	SAMR	A9	7
	CNCA	A12	7
	MARA	A13	7
	MNR	A7	6
	MOHURD	A8	5
Scatter Type	NATCM	A11	4
	ACWF	B2	4
	MOE	A4	3
	MOF	A5	2
	MHRSS	A6	2
	MEM	A19	1
	CCCYL	B1	1
	PBC	A15	0
	CSRC	A18	0
	RCS	B3	0

**Table 3:** The in-degree centrality values of different nodes.

Category	Government Node	Coding	In-Degree Centrality
Wide-range Connection Type	MCA	A2	16
	NHC	A1	14
	NDRC	A3	11
	MOE	A4	11
Small-range Connection Type	MOF	A5	9
	MHRSS	A6	7
	NHSA	A10	7
	CNCA	A12	7
	MOHURD	A8	6
	CBIRC	A17	6
	MNR	A7	5
	SAMR	A9	5
	NATCM	A11	5
	MARA	A13	5
	MIIT	A14	5
	PBC	A15	5
Scatter Type	STA	A16	4
	MEM	A19	3
	CCCYL	B1	3
	ACWF	B2	3
	CSRC	A18	2
	RCS	B3	2

**Table 4:** The related calculation results of the index weight.

Index	Information Entropy	Information Utility	Index Weight
The main policies	0.9921	0.0079	0.2430
The medical resources	0.9951	0.0049	0.1508
The natural resources	0.9949	0.0051	0.1571
The economic support	0.9939	0.0061	0.1892
The personnel training and employment encouragement	0.9916	0.0084	0.2599

**Table 5:** The related calculation results of the node weight.

Government Node	Distance from the Best Solution	Distance from the Worst Solution	Relative Closeness	Node Weight
NHC	0.159	0.384	0.707	0.188
MCA	0.254	0.344	0.575	0.153
MOF	0.371	0.200	0.350	0.093
MNR	0.404	0.165	0.290	0.077
MOE	0.355	0.144	0.288	0.077
NHSA	0.396	0.152	0.277	0.074
MHRSS	0.380	0.097	0.204	0.054
NDRC	0.391	0.090	0.187	0.050
MIIT	0.406	0.082	0.169	0.045
NATCM	0.416	0.080	0.161	0.043
PBC	0.431	0.082	0.161	0.043
SAMR	0.428	0.038	0.081	0.022
STA	0.444	0.025	0.054	0.014
MARA	0.446	0.023	0.049	0.013
MOHURD	0.444	0.022	0.046	0.012
CNCA	0.446	0.020	0.044	0.012
MEM	0.448	0.019	0.040	0.011
ACWF	0.451	0.011	0.023	0.006
CBIRC	0.452	0.010	0.022	0.006
CCCYL	0.451	0.010	0.021	0.006
CSRC	0.456	0.006	0.013	0.004
RCS	0.458	0.001	0.003	0.001

**Table 6:** The results of the collaboration coverage and the collaboration structure entropy.

Rank	Government Node	Coding	Collaboration Coverage	Collaboration Structure Entropy
1	MIIT	A14	43.66	237.84
2	NHC	A1	42.58	230.48
3	NATCM	A11	12.73	46.73
4	MCA	A2	10.97	37.89
5	CNCA	A12	10.93	37.71
6	CBIRC	A17	10.88	37.49
7	NDRC	A3	4.95	11.44
8	MOE	A4	4.85	11.06
9	MEM	A19	4.64	10.26
10	MNR	A7	3.57	6.55
11	MOF	A5	3.22	5.42
12	NHSA	A10	2.94	4.56
13	MARA	A13	2.13	2.33
14	SAMR	A9	2.11	2.26
15	MOHURD	A8	1.76	1.44
16	MHRSS	A6	1.70	1.31
17	STA	A16	1.57	1.01
18	ACWF	B2	1.45	0.77
19	PBC	A15	1.00	0
20	CSRC	A18	1.00	0
21	CCCYL	B1	1.00	0
22	ACWF	B3	1.00	0

**Table 7:** The top 10 collaboration effect values and the corresponding node-pairs (retaining three decimal places).

Rank	Node-Pair	Collaboration Effect
1	A1–A14	84.572%
2	A11–A14	20.275%
3	A1–A11	19.648%
4	A2–A14	16.440%
5	A12–A14	16.361%
6	A14–A17	16.268%
7	A1–A2	15.931%
8	A1–A12	15.855%
9	A1–A17	15.763%
10	A3–A14	4.964%

## Data Availability

The data presented in this study are available upon request from the corresponding author.
